# IS BEING HOME GOOD FOR YOUR HEALTH? OUTCOMES OF MEDICAID HOME- AND COMMUNITY-BASED SERVICES

**DOI:** 10.1093/geroni/igz038.2017

**Published:** 2019-11-08

**Authors:** R Tamara Konetzka, Rebecca J Gorges, Daniel H Jung, Prachi H Sanghavi

**Affiliations:** University of Chicago, Chicago, Illinois, United States

## Abstract

Expanding home- and community-based services (HCBS) alternatives to nursing homes has become a priority for many state Medicaid programs. Dramatic policy changes affecting diverse patient and family populations are being made on the basis of surprisingly little evidence about the outcomes of HCBS relative to the alternatives. Our study is designed to address that gap, providing the first national estimates of plausibly causal health outcomes for recipients of Medicaid HCBS relative to nursing home care. We also examine heterogeneity of these effects by several important subgroups. Results indicate that HCBS users have rates of hospitalization similar to their nursing home counterparts, and rates are higher for those with dementia. Furthermore, disparities by race and ethnicity that have been well-documented in nursing homes also appear to extend to the HCBS setting. The costs of potentially poor outcomes need to be part of the policy equation.

